# Novel Selective Detection Method of Tumor Angiogenesis Factors Using Living Nano-Robots

**DOI:** 10.3390/s17071580

**Published:** 2017-07-14

**Authors:** Mohamed Al-Fandi, Nida Alshraiedeh, Rami Owies, Hala Alshdaifat, Omamah Al-Mahaseneh, Khadijah Al-Tall, Rawan Alawneh

**Affiliations:** 1 Nanotechnology Institute, Jordan University of Science and Technology, P.O. Box 3030, Irbid 22110, Jordan; oweis@just.edu.jo; 2 Faculty of Pharmacy, Jordan University of Science and Technology, P.O. Box 3030, Irbid 22110, Jordan; nhalshraiedeh@just.edu.jo; 3 Micro and Nano Systems Lab., Jordan University of Science and Technology, P.O. Box 3030, Irbid 22110, Jordan; halla.shdefaty@yahoo.com (H.A.); engomamah@yahoo.com (O.A.-M.); Altall@yahoo.com (K.A.-T.); rawan-jo@hotmail.com (R.A.)

**Keywords:** bio-nanosensor, bacteria, nano-robot, chemotaxis, cancer therapy, microfluidics

## Abstract

This paper reports a novel self-detection method for tumor cells using living nano-robots. These living robots are a nonpathogenic strain of *E. coli* bacteria equipped with naturally synthesized bio-nano-sensory systems that have an affinity to VEGF, an angiogenic factor overly-expressed by cancer cells. The VEGF-affinity/chemotaxis was assessed using several assays including the capillary chemotaxis assay, chemotaxis assay on soft agar, and chemotaxis assay on solid agar. In addition, a microfluidic device was developed to possibly discover tumor cells through the overexpressed vascular endothelial growth factor (VEGF). Various experiments to study the sensing characteristic of the nano-robots presented a strong response toward the VEGF. Thus, a new paradigm of selective targeting therapies for cancer can be advanced using swimming *E. coli* as self-navigator miniaturized robots as well as drug-delivery vehicles.

## 1. Introduction

Cancer is one of the leading causes of death globally. According to the World Health Organization, nearly one in six deaths is caused by cancer [1]. There are many types of conventional cancer treatments available such as surgery, radiotherapy, and chemotherapy [2]. However, they are associated with high toxicity and disadvantageous effects on normal healthy cells due to poor selectivity [2]. Therefore, developing a new specific treatment in which the drug will selectively attack cancerous cells is highly demanded. The targeted therapy will reduce the required drug dose along with the drug side effects. Moreover, the targeted therapy will also assist in delivering the drug to metastasized cells that are not easily detected [3]. Development of such efficient drug-delivery systems to adequately transport the therapeutic agents directly to the cancerous cells is very challenging. Due to the remarkable development in nanotechnology, new diagnostic, therapeutic, and tissue engineering approaches have been developed. Likewise, research on molecular communication and biological nanomachines as targeting drug delivery systems to specific cell in a human body has started [4,5,6]. Molecular communication has emerged as a communication scheme between bio-nanomachines—naturally exist or biologically synthesized—and biological medium using various types of molecules over a minute distance [7,8]. One of the promising approaches in this research area is the use of motile bacteria as nano-robots that will selectively deliver the drug to the desired target. Several studies used moving bacteria such as *Bifidobacteria*, *Salmonella*, *Escherichia coli (E. coli)*, *Vibrio cholera*, and *Listeria monocytogenes* as emerging vehicles or even vectors for genes [9,10,11]. Flagellated bacteria are the smallest and simplest free-living organisms. They are comprised of various length scales, spanning from tens of nanometers in their flagellar motors and sensors to a few microns in their cell-body. Despite their diminutive dimensions, and the fact that they consist of a single cell, bacteria are well-studied microorganisms. *E. coli* is being used frequently as a simple model to study chemotaxis of flagellated bacteria. *E. coli* bacteria are extremely sensitive to chemical attractants, and their sensitivity covers a wide range of concentrations that is about four orders of magnitude (μM → mM) [12]. Al-Fandi et al. developed a platform to study the swimming behavior of the nonpathogenic *E. coli* as nano-robots sensitive to the chemical gradients in their medium [11].

Many tissue and angiogenesis factors, such as vascular endothelial growth factor (VEGF) and basic fibroblast growth factor (bFGF), are overexpressed by cancerous cells and have been suggested for their role in cancer pathogenicity [13]. VEGF, also known as the vascular permeability factor (VPF), is a potent angiogenic factor essential for the formation of new blood vessels from pre-existing vasculature. Angiogenesis is very important in normal physiological processes such as wound healing, hematopoiesis, and organ regeneration [13,14,15]. According to the US National Cancer Institute, tumor angiogenesis is the proliferation of a network of blood vessels that penetrates into cancerous growths, supplying nutrients and oxygen and removing waste products [12]. This process starts with cancerous tumor cells releasing molecules that constantly send signals to the surrounding normal host tissue. This chemical signaling activates certain genes in the host tissue that, in turn, make proteins to encourage the growth of new blood vessels [2]. Since this chemical signal attracts blood vessels, most likely it may have a certain influence on the *E. coli* nano-sensory system. Consequently, these chemical factors have the potential to affect the movement of the bacterial nano-robots.

This study illustrated that VEGF is a chemoattractant to *E. coli*. VEGF is overly expressed in cancer cells; therefore, it can be investigated in the future if this method can be applied to detect cancer cells. Furthermore, *E. coli* could be used as bio-nano-robots for targeted drug-delivery. The bacterial chemotaxis toward VEGF was assessed using several assays including capillary chemotaxis assay, chemotaxis assay on soft agar, and chemotaxis assay on solid agar. In addition, a microfluidic device was developed to generate a stable concentration gradient of VEFG to monitor the locomotion of nonpathogenic *E. coli* (AW405) in response to the applied gradient.

## 2. Material and Methods

Glucose and nickel were obtained from ACROS ORGANICS (Geel, Belgium). VEGF was obtained from PEPROTECH (Rocky Hill, NJ, USA). All chemicals were dissolved in deionized (DI) water. Water was chosen as a solvent per the recommendation of the VEGF manufacturing protocol. Glucose and nickel were tested over the range 10^−7^ to 10^−1^ M and 3 mg/1 mL was chosen as a threshold concentration according to the results.

VEGF preparation: A vial of 10 μg of VEGF was cooled down to room temperature then centrifuged prior to opening. After centrifugation, the VEGF was reconstituted in water to a concentration of 1.0 mg/mL without a vortex.

### 2.1. Living Nanorobots Preparation

Flagellar bacteria such as *E. coli* were utilized as nano-bio-robots. These robots have an integrated system equipped with precise nano-sensory elements which controls their propulsion system according to the chemical gradients in their medium. The preparation of these living, motile robots is simple and can be carried out without the need of sophisticated nanofabrication facilities. At first, Luria-Bertani LB broth (1 g Tryptone, 1 g Sodium chloride, 0.5 g Yeast Extract in 100 mL deionized water) was prepared, sterilized, and inoculated with a fresh growth of *E. coli* (AW405) obtained from American Type Culture Collection (ATCC) (Manassas, VA, USA). The culture tubes were incubated for 4 h at 37 °C with agitation at 120 min^−1^. Mid-log, *E. coli* was centrifuged at 4000 rpm, 4 °C for 10 min, and then resuspended in sterile phosphate buffered saline (PBS) motility buffer (H2O, 10 mM K2HPO4/KH2PO4, 70 mM NaCl, 0.1 mM EDTA, pH 7.0) to reach an optical density (OD550) equivalent to 5 × 10^7^ CFU/mL.

### 2.2. Chemotaxis Assay on Soft Agar

The chemotaxis assay on soft agar was prepared according to the procedures followed by Hazeleger et al. (1998) [16]. Concisely, 1 mL of the bacterial culture concentrate was mixed with 14 mL of 0.3% molten soft agar then poured into a sterile Petri plate. The agar was left at room temperature to solidify. Sterile filter paper discs were impregnated with 10 μL of the peak concentration of each chemical including glucose, nickel, water, and VEGF. Then they were placed on top of the solidified bacterial cell-agar mixture in the of the Petri dishes. The plates were incubated at 37 °C overnight. Water was used as a control. The agar mixture was observed for the formation of a turbid zone around the disc. The experiment was duplicated for each chemo effector.

### 2.3. Chemotaxis Assay on Solid Agar

A total of 500 μL of bacterial culture concentrate was evenly spread over 1.5% solid agar surface. A sterile filter paper disc was impregnated with 10 μL of VEGF (1.0 mg/mL) and positioned on top of the solid agar mixture with the bacteria. Ten μL of water was used as a control in this assay. The plates were incubated at 37 °C overnight. The top surface of agar was observed for the formation of a turbid zone around the disc. To confirm the influence each chemotaxis substance, this experiment was executed six times.

### 2.4. Capillary Chemotaxis Assays

Capillary chemotaxis assay was done according to the procedures described by Adler [17] with some modifications. Capillary tubes filled with 5 μL of peak concentration of glucose (known chemoattractant), DI water (solvent), and VEGF (potential chemoattractant). The other end of capillary tubes was sealed. Then the open end of the capillary was placed into 1 mL of bacterial-cell suspension for 60 min. After incubation for 1 h, the capillary was removed and the exterior was washed gently with a thin stream of PBS. The sealed end was opened and the liquid inside the capillary was poured into a well of a 96-well plate containing 180 μL PBS. Serial 10-fold dilution of the recovered liquid was performed in a 96-well microliter plate using sterile PBS to quantify the number of cells that entered the capillary tubes. Then three aliquots (10 μL each) from each well were spotted on the surface of LBA and incubated at 37 °C. After 24 h, the number of colonies from each spot were counted. Each experiment was done at least in duplicate.

### 2.5. Chemotaxis Assay Using Fabricated Microfluidic Chip

**Figure 1:**
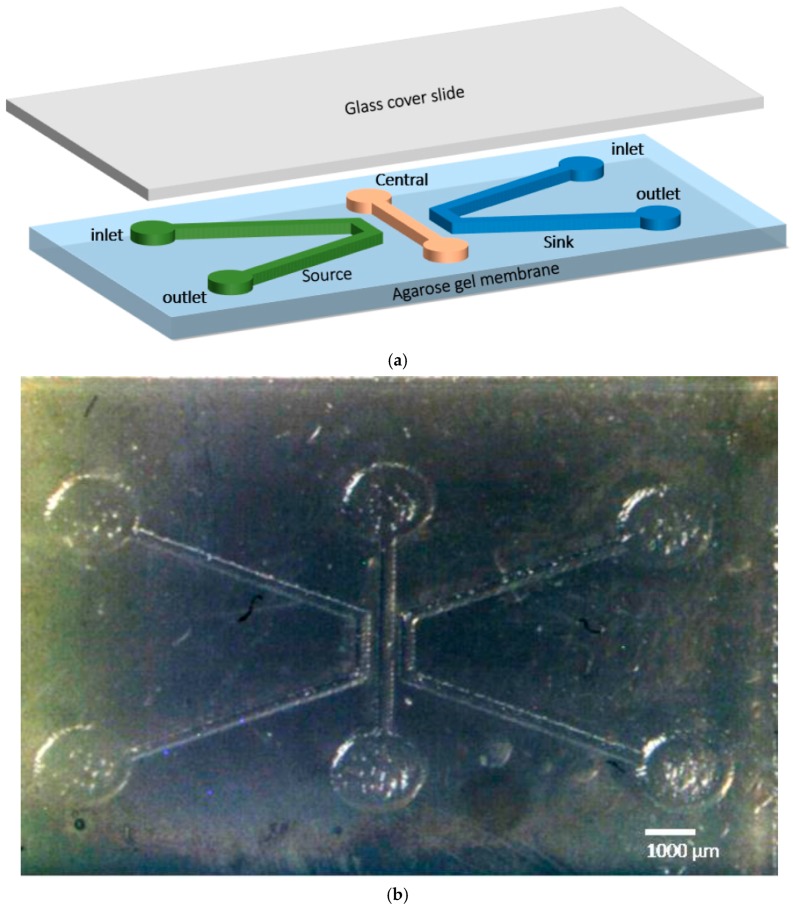
(**a**) Schematic of the microfluidic chip for motility chemotaxis; (**b**) Optical image of the chemotaxis microfluidic chip using agarose gel (source, central, sink channels).

To investigate the motility chemotaxis of the living nano-robots, a microfluidic chip layout was planned using porous agarose gel. The design of the microfluidic chip is illustrated in Figure 1a. The chip is composed of three parallel channels—source, sink, and central channel—fabricated from a piece of (22 × 15 mm, with a 6 mm thickness) agarose gel. All dimensions of the microfluidic channels were carefully optimized after a comprehensive study on the diffusion time of the chemo-effector substance. Furthermore, the chemotactic response of *E. coli* was evaluated and simulated in a previous study [18]. The microfluidic chip configuration was printed on a shrinking paper, then it was heated in an oven at 150 °C for 3 min, the size of the printed chip shrunk about 60% from its original size. The pattern on the shrunk paper was transferred into the solid agarose gel, as seen in Figure 1b. Three different concentrations (1, 2, 3%) of agarose gel were tested for diffusion of the chemoattractant material. The agarose gel was prepared by mixing 1, 2, and 3 g of agarose powder with 10 mL TAE buffer (solution contains .04 M Tris base, .04 M acetate, and .001 M EDTA) to achieve 1, 2, and 3% agarose gel, respectively. The mixture was heated on a hotplate at 250 °C for 5 min. Subsequently, the mixture was poured above the patterned shrunk paper in a petri dish and set at room temperature for 30 min until it was completely solidified. The inlet and outlet ports of the source, sink, and central channel were punched using a biopsy puncher (2 mm Miltex Biopsy punch with Plunger) where chemo effectors (glucose or VEGF), water and bacteria cells can be loaded, respectively.

Ten μL of glucose (D- Glucose monohydrate) solution (3 mg/1 mL) or VEGF (10 μg /10 μL) and 10 μL of water were injected in the source and sink channels’ reservoirs, respectively, and left for about 5 min to generate a stable gradient of glucose or VEGF across the central channel. Then, 5 μL of motility buffer PBS was injected into the central channel followed by the injection of 10 μL of *E. coli* cells. PDMS blockage was used to seal the inlet and outlet of the central channel, thereby preventing the leakage of the medium as well as allowing self-movement of the *E. coli* nano-robots.

A differential interference contrast (DIC) inverted microscope (Nikon Eclipse Ti- E) was used to capture images and videos of the *E. coli* response to the applied gradients of chemo effectors. Then Image J software (developed by the Research Services Branch, National Institute of Mental Health, Bethesda, MD, USA) was used for image processing and for distribution analysis across the central channel.

## 3. Results and Discussion

According to angiogenesis science, tumor cells at early stages stimulate blood vessels to grow toward the tumor [2,19]. This process occurs when tumor cells transmit chemical signals, such as VEGF, to attract the growth of the blood vessels which supply the nutritive requirements for the tumor cells. Swimming non-pathogenic *E. coli* (living nanorobots) were investigated for VEGF chemotaxis. This can be helpful in intercepting this signal, and possibly detecting cancer in its early stages by exploiting this/such chemotaxis. We used *E. coli* because it has the ability to distinguish its surrounding environment by its chemoreceptors (methyl-accepting chemotaxis proteins: MCPs) and demonstrates a directional movement toward a chemo-effector via nano flagellar motors [20,21,22].

### 3.1. Growth VEGF Chemotaxis

**Figure 2:**
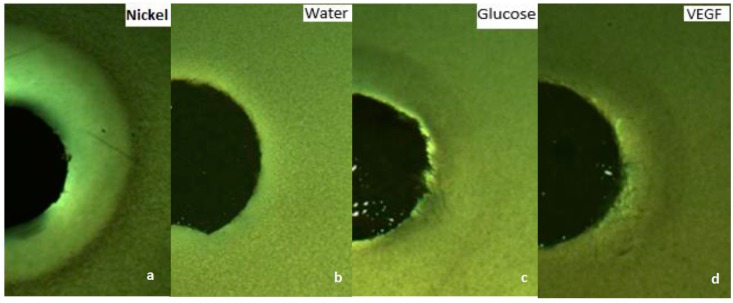
Chemotaxis assay using the disc method on soft agar at (10×) magnification, (**a**) Nickel (chemo repellant): a clear zone was formed around the disc; (**b**) Water was used as control, it elicits no chemotactic responses from *E. coli*; (**c**) Glucose (chemoattractant): a turbid zone was formed; (**d**) VEGF (potential chemo attractant): a turbid zone was formed.

**Figure 3:**
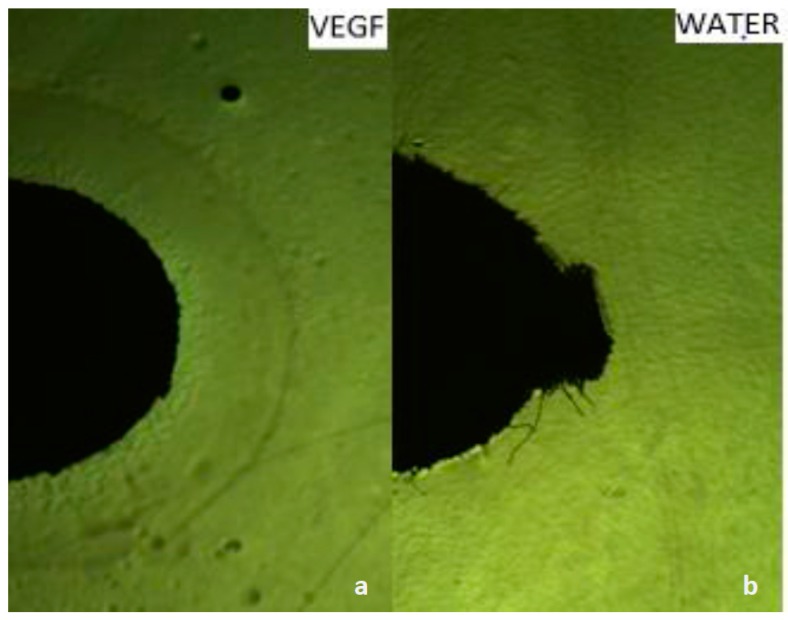
Chemotaxis assay using the disc method on solid agar at (10×) magnification, (**a**) VEGF (potential chemoattractant): a dense ring was formed; and (**b**) water used as control elicited no chemotactic responses from *E. coli*.

To confirm the chemotaxis performance of the nanorobots toward VEGF, several assays were conducted including chemotaxis-growth assays on soft and solid agar as well as motility chemotaxis assays using capillaries and fabricated microfluidic chips. In the soft agar growth assay, we confirmed our species chemotaxis by stabbing bacterial cultures grown on a soft LB agar. After overnight incubation, the *E. coli* was diffused throughout the medium, giving a hazy turbid look. Subsequently, we evaluated the chemotaxis behavior of *E. coli* grown on 0.3% soft agar toward glucose (known chemoattractant), nickel (known chemorepellent), VEGF (potential chemoattractant) impregnated on paper discs, and finally water as a negative control. After overnight incubation, turbid rings of *E. coli* formed around the disc impregnated with glucose as well as VEGF (Figure 2c,d). However, a clear zone formed around the disc impregnated with nickel as seen in Figure 2a. While water showed no effect on the growth of *E. coli* (Figure 2b). Therefore, the overall performance of *E. coli* growth showed a chemoattractant behavior toward VEGF. Furthermore, we also qualitatively confirmed the chemotaxis-growth behavior of *E. coli* toward VEGF by placing impregnated discs with VEGF and water on a solid agar streaked with *E. coli*. A circular dense ring around the disc impregnated with VEGF was formed (Figure 3a) whereas water, that was used as a control, elicited no chemotactic responses from *E. coli* as shown in Figure 3b.

### 3.2. Motility VEGF Chemotaxis

**Figure 4:**
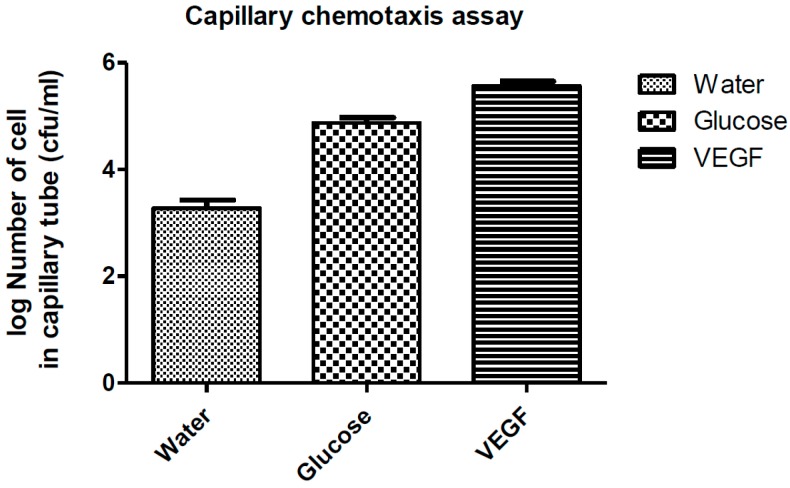
*E. coli* chemotaxis toward water, glucose, and VEGF by capillary assay.

Both previously used growth assays qualitatively confirmed that VEGF is a chemoattractant constituent to *E. coli*. Recently, a capillary chemotaxis assay was used to evaluate chemotaxis in several organisms such as camp. *Jejuni*, camp. *Concisus*, and *E. coli* [12,23,24]. In our study, we also utilized this capillary chemotaxis motility assay to quantitatively determine chemotaxis in *E. coli*; chemotaxis was expressed as the ratio of the number of cells in the capillary with the attractant substance to the number of cells in a control capillary without the attractant as described by Paster [24]. If the ratio of bacteria cells that entered the capillary tube filled with a chemical compared to the number of bacteria cells that entered the chemical free capillary tube is greater than 1, then the chemical is considered a chemoattractant ingredient [25]. In our experiment, the number of bacterial cells that entered the capillary tube that filled with 5 μL VEGF is significantly higher than that filled with water (*p* < 0.005) as shown in Figure 4. This finding quantitatively confirmed that VEGF is a significant chemoattractant constituent to *E. coli*.

**Figure 5:**
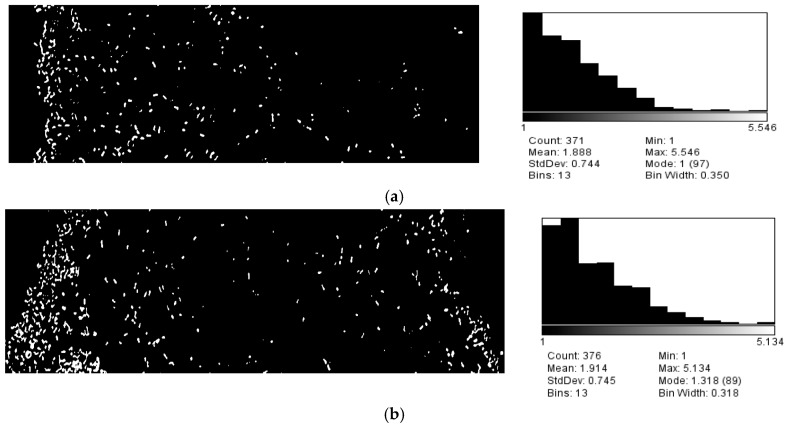
(**a**) Chemotaxis assay using fabricated microfluidic chip at (400×) magnification. Bacterial response to the glucose gradient at the central channel, the left channel of the chip was filled with 10 μL glucose solution (3 mg/mL), the right channel was filled with 10 μL deionized water. The histogram represents the cell distribution across the bacterial channel versus the position in the channel, the distribution values showed bias toward the highest concentration of glucose. The distribution of bacteria across its channel was analyzed using Image J software. (**b**) Chemotaxis assay using fabricated microfluidic chip at (400×) magnification. Bacterial response to the VEGF gradient at the central channel, the left channel of the chip was filled with 10 μL VEGF solution (10 μg/10 μL), the right channel was filled with 10 μL buffer solution (deionized water). The histogram represents the cell distribution across the bacterial channel versus the position in the channel, the distribution values showed bias toward the highest concentration of VEGF.

We also developed a prototype of a three-channel microfluidic device that enabled us to study the response and swimming behavior of motile living nanorobots under different chemoattractant gradients. The model of three parallel channels allows fast execution, has a low fabrication cost, and can establish a stable chemical gradient, which made it a suitable design for our work. Agarose gel is a three-dimensional structure with pores that permit chemoattractant substances and water to diffuse through, generating a stable concentration gradient across the central channel where the bacterial response can be monitored. It is worth mentioning that the diffusion time was appraised for different agarose concentrations (1, 2, and 3%) by injecting three red drops of ink in three identical holes. The total diffusion time to establish a stable gradient across the chip was evaluated experimentally as well as analytically. The diffusion time across agarose gel was estimated and optimized experimentally. Several holes were punched in the center of three separated layers of 1, 2, and 3% agarose gel and the diffusion of the ink was observed for 1 h. The diameter of the diffusion rings—which were formed around the holes—were measured to evaluate the diffusion rate. As a result, 1% gel concentration showed the fastest diffusion and was exploited to design the microfluidic chip. The diffusion time to reach a stable gradient of VEGF across the central channel was analytically estimated. According to Fick’s Law, the diffusion time of the freely-diffused particles due to Brownian motion can be calculated using t_d_ = (L_d_)^2^/4D, where L_d_ is the diffusion length and D is the diffusion coefficient. From a previous study the diffusion time for the VEGF was taken to be 5.87 × 10^−7^ cm^2^ s^−1^ [26]. Using the width of the fabricated central channel—which is the diffusion length—to be 400 μm then the diffusion time across the central channel is about 11 min. Therefore, the total diffusion time—which is the diffusion time across agarose gel and the central channel, to reach a stable gradient of VEGF—was estimated at about 16 min. This total diffusion time to attain a steady state flux of the chemo-effector was sufficient to study the chemotaxis behavior of *E. coli* in response to VEGF. So, when a stable concentration gradient of chemo-effectors (glucose or VEGF) was established across the source channel while water gradient across the sink channel, highly motile *E. coli* cells were injected into the inlet reservoir of the central channel. The swimming behavior of *E. coli* living robots was observed using a DIC optical microscope and images were then analyzed using Image J. Figure 5a shows that the living robots were attracted adjacent to the side where the glucose diffuses. Likewise, as can be seen in Figure 5b, the migration velocity toward VEGF was higher than the water side. These findings indicate that glucose and VEGF have chemoattractant influence on the motile *E. coli* bacteria (Figure 5a,b).

Finally, these promising findings demonstrate that *E. coli* has significant chemo-attracting properties in the direction of the overexpressed angiogenesis factors VEGF produced by tumors. Therefore, *E. coli* swimming nanorobots can be considered for use as self-locating drug-delivery systems to precisely targeting tumor cells.

## 4. Conclusions

In our work, we demonstrated a novel method to consider swimming *E. coli* nanorobots for early detection of tumor cells, and possibly for drug-delivery systems. The chemotaxis response of *E. coli* cells proves the ability of these cells to work as diminutive robots that can identify the VEGF, which is overexpressed by tumor cells more than normal cells. This promising finding will help in developing a targeted therapy practice for cancer by using *E. coli* as microscopic robots loaded with chemotherapeutic agents.
