# Erratum: Al-Fandi, M.; et al. Novel Selective Detection Method of Tumor Angiogenesis Factors Using Living Nano-Robots. *Sensors* 2017, *17*, 1580

**DOI:** 10.3390/s17112492

**Published:** 2017-10-30

**Authors:** Mohamed Al-Fandi, Nida Alshraiedeh, Rami Oweis, Hala Alshdaifat, Omamah Al-Mahaseneh, Khadijah Al-Tall, Rawan Alawneh

**Affiliations:** 1 Nanotechnology Institute, Jordan University of Science and Technology, P.O. Box 3030, Irbid 22110, Jordan; oweis@just.edu.jo; 2 Faculty of Pharmacy, Jordan University of Science and Technology, P.O. Box 3030, Irbid 22110, Jordan; nhalshraiedeh@just.edu.jo; 3 Micro and Nano Systems Lab., Jordan University of Science and Technology, P.O. Box 3030, Irbid 22110, Jordan; halla.shdefaty@yahoo.com (H.A.); engomamah@yahoo.com (O.A.-M.); Altall@yahoo.com (K.A.-T.); rawan-jo@hotmail.com (R.A.)

The authors wish to correct the spelling of the third author’s name from Rami Owies to Rami Oweis in their paper published in *Sensors* [1], doi:10.3390/s17071580, http://www.mdpi.com/1424-8220/17/7/1580. The manuscript will be updated and the original will remain online on the article webpage, with a reference to this Erratum.
